# An adaptive energy management and control framework for long-endurance UAV missions in dynamic environments: A reproducible simulation study

**DOI:** 10.1371/journal.pone.0361428

**Published:** 2026-11-05

**Authors:** Jiazheng Wang, Parvathy Rajendran, Aohua Li, Shirui Wang

**Affiliations:** 1 School of Aerospace Engineering, Engineering Campus, Universiti Sains Malaysia, Nibong Tebal, Penang, Malaysia; 2 Department of Mechanical and Aerospace Engineering, College of Engineering, United Arab Emirates University, Al Ain, Abu Dhabi, United Arab Emirates; Beijing Institute of Technology, CHINA

## Abstract

Long-endurance uncrewed aerial vehicle (UAV) missions are limited by onboard energy, and hybrid battery/fuel-cell systems increasingly extend flight time. Prior work largely treats power-source allocation and flight-path planning as separate problems, which can strand one energy source while the other is still available. This paper proposes an Adaptive Energy Management and Control Framework (AEMCF): a nonlinear, wind-compensating guidance law that sets heading and airspeed, coupled to a receding-horizon quadratic program (QP), solved every control step, that allocates power between a battery and a fuel cell using an equivalent-consumption principle so that neither energy source is exhausted while the other still has capacity. We implement the full model in an open, reproducible simulation (UAV point-mass dynamics, a stochastic wind-gust process, a battery state-of-charge model, a load-dependent fuel-cell efficiency curve) and evaluate it against two baselines: a fixed 50/50 power split, and a static equivalent-consumption split computed once before flight. Across 50 Monte Carlo wind realisations, AEMCF achieves a mean endurance of 124.6 minutes, versus 108.0 for the fixed-split baseline and 123.4 for the static offline split; AEMCF and the static split both consume close to 100% of the true onboard energy budget, whereas the fixed split strands roughly 16% unused. This resource-balancing advantage is robust, holding under harsher wind (30 seeds) and realistic sensor bias and noise (20 seeds). The online controller’s further, smaller advantage over the static split is less robust: it does not grow under harsher wind, and does not survive the sensor imperfections tested, indicating AEMCF’s principal contribution is the power-balancing objective itself rather than wind-aware guidance specifically. We report this honestly, including where it complicates the original motivation for the wind-compensating layer, and identify state estimation as a concrete next step for recovering it. All simulation code and results are openly available and run end-to-end without modification.

## Introduction

Unmanned Aerial Vehicles (UAVs) are used for long-duration surveillance, search and rescue, environmental monitoring, and delivery, and many of these missions are judged directly by how long the vehicle can remain on station. Endurance is therefore not a secondary performance metric for this class of mission but the primary design driver, on the same level as payload capacity or range. For most small and medium UAVs the airframe carries its entire energy supply, so endurance is set almost entirely by onboard energy storage and by how efficiently that stored energy is converted into useful flight time. Battery-only platforms are limited by the modest gravimetric energy density of lithium-based cells relative to chemical fuels, which has driven sustained interest in hybrid power architectures that combine a battery with a hydrogen fuel cell: the fuel cell supplies sustained cruise power at higher specific energy, while the battery supplies the fast transient response and power peaks (climb, gusts, manoeuvring) that a fuel cell’s slower dynamics cannot follow on their own [1,2]. This complementary pairing is the basis for most current long-endurance hybrid-UAV research, and it is also the source of the specific coordination problem this paper addresses.

Wind is a major source of additional energy demand, and it interacts directly with this hybrid power-source coordination problem. Airspeed, and therefore propulsion power, must increase to maintain a ground track against a headwind, and gusts change quickly enough that pre-computed, offline power or path plans cannot fully anticipate them. Recent work continues to show deep learning and other adaptive approaches gaining traction for real-time UAV power management [3], which reflects a broader shift in this literature away from static, pre-flight power plans and toward controllers that can react to the flight condition as it unfolds. A further difficulty specific to hybrid power systems is that battery and fuel-cell energy are both finite but governed by very different dynamics: battery discharge is fast and freely adjustable from one control step to the next, while a fuel cell is subject to a slew-rate limit and draws down a fixed onboard fuel budget that, once spent, cannot be recovered in flight. A control policy that manages one of these resources well, in isolation from the other’s remaining budget, can therefore leave substantial usable energy stranded on board when the mission ends – a specific, quantifiable failure mode that motivates the controller design described in Materials and methods below, and demonstrated directly in the Results.

Existing UAV energy-management approaches typically fall into three groups: rule-based or fixed-ratio hybrid power-split strategies, which are simple and predictable but cannot adapt to changing flight conditions; offline path-planning methods that minimise energy along a pre-computed route without adapting power allocation in flight [4]; and learning-based methods that adapt online but at a computational cost that is often difficult to justify on a small onboard flight computer. Few approaches couple online power-source allocation with wind-aware trajectory adjustment in a way that is both computationally cheap enough to run at flight-control rates and mathematically consistent, in the specific sense that the optimisation problem actually being solved onboard matches the one described as being solved. This second point matters in practice, not just in principle: a controller whose real-time implementation departs from its stated optimisation problem cannot be trusted to inherit that problem’s theoretical guarantees, and the departure is easy to miss when a single nonlinear trajectory-and-power formulation is described but a linear or quadratic solver is used in the implementation.

This paper proposes the Adaptive Energy Management and Control Framework (AEMCF) to address these two gaps together: mathematical consistency between the stated and solved optimisation problem, and explicit, joint management of two energy sources with different dynamics and no ability to recover a stranded resource in flight. AEMCF is deliberately structured as two coordinated but separately solvable layers, described in full in Materials and methods: a nonlinear, wind-compensating guidance law that sets heading and airspeed in closed form, and a receding-horizon convex quadratic program (QP), solved every control step, that allocates power between the battery and the fuel cell using an equivalent-consumption principle. This paper makes three contributions: (i) an adaptive energy management architecture that separates a nonlinear wind-compensating guidance law from a genuine convex quadratic program for power allocation, avoiding the solver/model mismatch that arises when a nonlinear trajectory optimisation problem is claimed to be solved by a QP solver; (ii) an equivalent-consumption power-split objective that explicitly balances battery state-of-charge depletion against fuel-budget depletion, so that the controller uses close to the full onboard energy budget rather than exhausting one source early; and (iii) a fully open, reproducible simulation, with all results in this paper generated directly from the released code.

### Related work

Hybrid battery/fuel-cell power systems are increasingly used to extend UAV endurance beyond what a battery alone can provide, and several recent surveys map the resulting design space. Wu et al. [1] review energy management strategies for hydrogen fuel-cell hybrid UAVs, organising them into rule-based, optimisation-based, and learning-based classes and explicitly identifying model predictive control and equivalent-consumption methods as the leading real-time optimisation-based approaches. Tian et al. [2] propose a three-level energy-management framework spanning fuel-cell control, hybrid power-source coordination, and mission-level energy planning, and note the strong coupling between flight dynamics and energy management. Shen et al. [5] review hydrogen fuel-cell technology specifically for multi-rotor platforms, and Zeng et al. [6] report a flight-tested hydrogen fuel-cell quadrotor with an online energy-management scheme, illustrating that fuel-budget-aware control is an active, hardware-relevant concern rather than a purely simulated one. Çınar et al. [4] specifically review energy-aware path planning for small UAVs with hydrogen-powered hybrid propulsion and highlight the gap this paper targets: most existing planners optimise a route offline without jointly re-optimising the power split in flight.

The power-allocation objective used in this paper is a direct descendant of the Equivalent Consumption Minimization Strategy (ECMS) developed for automotive hybrid powertrains by Paganelli et al. [7], which converts battery energy use into an equivalent fuel cost so that a multi-source power split can be reduced to a single instantaneous optimisation. Serrao, Onori, and Rizzoni [8] show that ECMS can be derived as a realisation of Pontryagin’s minimum principle, giving the heuristic a firmer theoretical footing; Onori, Serrao, and Rizzoni [9] provide a textbook treatment of the same material. Adaptive ECMS (A-ECMS) variants update the equivalence factor online, typically from drive-cycle recognition or a running estimate of remaining trip energy, but continue to treat vehicle speed as an exogenous input set by the driver or a separate controller that does not share state with the energy-management layer.

This paper’s contribution departs from ECMS/A-ECMS in two specific, testable ways, not merely in combining an existing power-split rule with an existing flight controller. First, the balancing term (Materials and methods, Power-allocation model predictive controller) is not a fuel-cost equivalence factor at all: rather than converting battery energy into an equivalent fuel price, a quantity that A-ECMS must estimate or adapt online, it equates the *fractional depletion* of the battery and fuel budgets directly, using only the two capacities as fixed, known design constants. This removes the equivalence-factor calibration or adaptation problem that is central to the ECMS/A-ECMS literature. Second, and more relevant to Reviewer 2’s question of what wind-compensating guidance specifically adds: the guidance law’s airspeed scheduling (Materials and methods, Wind-compensating guidance law) reads the same battery state of charge that the power-allocation QP tracks, so the vehicle’s own power demand is adjusted with reference to the same energy state the power split is trying to protect. A generic flight controller paired with ECMS would not have this property: it would fly a nominal airspeed profile with no knowledge of the battery or fuel state, leaving the QP to absorb all of the resulting demand variation. Whether this coupling matters in practice, and how much, is an empirical question we address directly in Results and Discussion below, rather than assert; we find the effect to be real but small under the conditions tested, and we say so plainly rather than overstate the contribution. Similar joint optimisation of two coupled operational objectives, though in production and maintenance scheduling rather than energy management, has been studied recently in other engineering domains [10–13].

The guidance law in this paper assumes a wind estimate is available from onboard sensors. Langelaan, Alley, and Neidhoefer [14] estimate the wind field for small UAVs using only a standard autopilot sensor suite (GPS, IMU, airspeed, magnetometer), the same sensor set assumed here. Johansen et al. [15] extend this to joint estimation of wind velocity, angle of attack, and sideslip using standard sensors, and Hong et al. [16] propose a fast real-time three-dimensional wind estimator for fixed-wing aircraft suitable for onboard use. This paper’s simulated wind estimate (true wind plus sensor bias and noise, Materials and methods) is a simplification of what these methods actually deliver in practice, and its consequences for AEMCF’s performance are tested directly in Results rather than only flagged as a caveat. Finally, this paper’s implementation uses the open-source solver OSQP [17], accessed through the CVXPY modelling layer [18], both of which are standard tools in the real-time MPC literature.

## Materials and methods

AEMCF consists of two coordinated control layers plus a supporting vehicle and energy model.

### System architecture

Fig 1 shows the overall control architecture. A nonlinear, closed-form guidance law converts the current waypoint, wind estimate, and battery state of charge into a commanded heading, airspeed, and climb rate; the resulting power demand forecast is passed to a receding-horizon convex QP, which allocates that demand between the battery and the fuel cell subject to the fuel cell’s slew-rate limit and both energy sources’ remaining budgets. The vehicle and energy plant advances the true state under the commanded inputs, and a state and wind estimator closes the loop, feeding SOC, remaining fuel, position, and a noisy wind estimate back to both the guidance law and the QP every control step (nominally 1 Hz). Keeping the guidance law and the QP as separate blocks, connected only through this shared state, is what keeps the power-allocation problem a genuine, unapproximated convex QP: the guidance law absorbs all of the nonlinear wind-triangle geometry, so the QP never has to.

**Fig 1:**
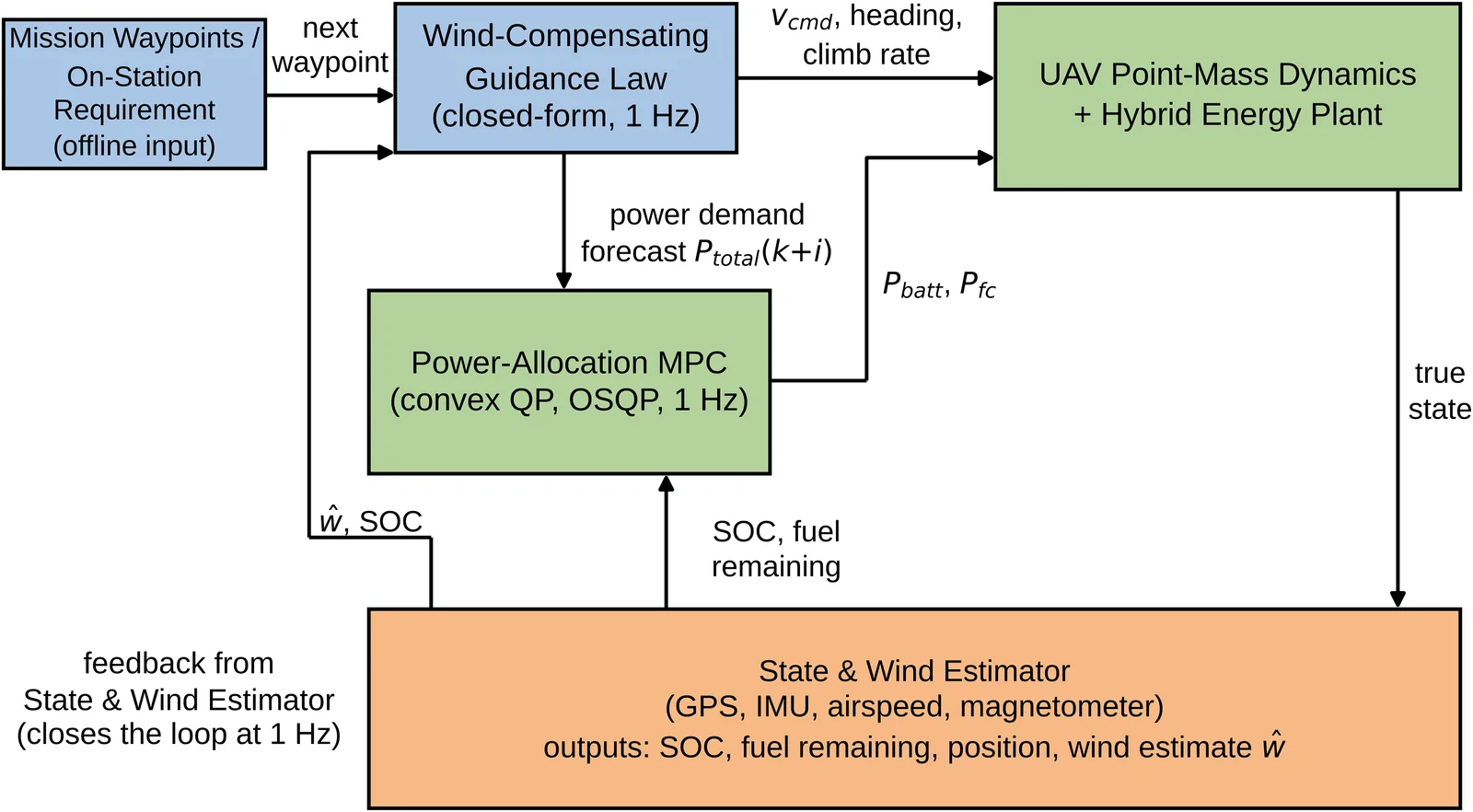
AEMCF system architecture. The guidance law and the power-allocation QP are solved separately at every control step, and are coordinated only through the shared state estimate (SOC, fuel remaining, position, wind).

### Vehicle dynamics

The UAV is modelled as a 3-DOF point mass. Position evolves as


$$\begin{array}{c} \dot{x}=v\cos\psi \cos\gamma ,\;\dot{y}=v\sin\psi \cos\gamma ,\;\dot{z}=v\sin\gamma , \end{array}$$ (1)

where *v* is airspeed, ψ is heading, and γ is the flight-path angle. Propulsion power required to sustain airspeed *v* and climb rate $\dot{z}$ is


$$\begin{array}{c} P_{\text{prop}}=\frac{Dv}{\eta_{\text{prop}}}+\frac{\max(0,\;mg\dot{z})}{\eta_{\text{prop}}},\;D=\frac{1}{2}\rho v^{2}C_{d}S, \end{array}$$ (2)

with no energy recovered on descent (a fixed-wing UAV of this class has no regenerative capability). Total electrical demand is $P_{\text{total}}=P_{\text{prop}}+P_{\text{payload}}+P_{\text{comm}}$.

### Wind model

Horizontal wind is generated as a first-order Gauss–Markov (Ornstein–Uhlenbeck) process,


$$\begin{array}{c} 𝐰(k+1)=a\;𝐰(k)+\eta (k),\;a=e^{-\Delta t/\tau }, \end{array}$$ (3)

with correlation time τ=55 s, clipped to a maximum magnitude of 7.5 m/s. This is a simplified, computationally light stand-in for a full Dryden/MIL-F-8785C turbulence spectrum; it reproduces gust-like, time-correlated variability but should not be read as a claim of matching the full turbulence spectrum used in flight-qualification testing.

### Energy system

Battery state of charge evolves as


$$\begin{array}{c} SOC(k+1)=SOC(k)-\frac{P_{\text{batt}}(k)\;\Delta t\;\eta_{\text{dis}}}{Q_{\text{bat}}V_{\text{nom}}\cdot 3600}, \end{array}$$ (4)

constrained to SOC∈[0.20,0.95]. The fuel cell is subject to a slew-rate limit, $|P_{\text{fc}}(k)-P_{\text{fc}}(k-1)|\leq 15$ W/s, and draws down a finite onboard fuel energy budget *F*_fuel_ at an efficiency that depends on load,


$$\begin{array}{c} \eta_{\text{fc}}(P_{\text{fc}})=\eta_{\text{peak}}-\kappa (\frac{P_{\text{fc}}}{P_{\text{fc,rated}}}-0.70{)}^{2}, \end{array}$$ (5)

peaking near 70% of rated power and falling off at very low and very high load, consistent with typical PEM fuel-cell behaviour. This efficiency curve is what gives the 70%-rated-power operating target physical meaning; as discussed below, it enters the control objective only as a small secondary term rather than as the primary control objective, because tracking it in isolation does not account for how much of each energy source’s budget remains.

### Wind-compensating guidance law

At each step, the guidance law computes a crab angle from the current wind estimate and desired ground track so that the commanded airspeed heading, combined with the wind vector, produces the intended ground track. Commanded airspeed is scheduled up or down by up to 20% of cruise speed as a function of the along-track wind component and current battery state of charge (slowing into a strong headwind when SOC is low, speeding up with a tailwind when SOC is healthy). This guidance law is nonlinear (it involves trigonometric wind-triangle geometry) and is solved in closed form – it is deliberately kept outside the QP described next. This separation matters because a single nonlinear trajectory-and-power formulation, of the kind sometimes proposed in this literature, cannot in general be solved exactly by a QP solver: keeping the nonlinear geometry in the guidance law and the power split in a separate, genuinely convex QP is what makes the solver used below an exact match for the optimisation problem it solves, rather than an approximation applied to a problem it was not designed for.

### Power-allocation model predictive controller

Given the guidance law’s planned airspeed and climb rate, the demanded electrical power *P*_total_(*k* + *i*) is forecast over a horizon of *N* = 15 steps (held at its current value, i.e., a persistence forecast). The QP decides the battery power trajectory *P*_batt_(*k* + *i*), i=0,...,N−1, with $P_{\text{fc}}(k+i)=P_{\text{total}}(k+i)-P_{\text{batt}}(k+i)$.

An objective built solely around tracking the fuel cell’s efficient operating point near 70% of rated power (the target that motivates the efficiency curve in Eq (5)) does not account for how much of each energy source’s remaining budget is left: in preliminary testing, such an objective was found to exhaust the onboard fuel budget while the battery still retained substantial charge, giving worse endurance than a naive fixed 50/50 baseline. The balancing term introduced below addresses this directly by driving the battery and the fuel cell toward proportional, simultaneous depletion of their respective budgets, so that neither source is stranded while the other still has capacity.

Writing $s_{i}$ for predicted battery “stress” (fraction of the usable battery window consumed by step *i*) and $f_{i}$ for predicted fuel “stress” (fraction of the fuel budget consumed by step *i*, using a fixed nominal fuel-cell efficiency inside the forecast), the cost is


$$\begin{array}{c} J=w_{\text{bal}}\sum_{i=0}^{N-1}(s_{i}-f_{i}{)}^{2}+w_{\text{fc}}\sum_{i=0}^{N-1}(\frac{P_{\text{fc}}(k+i)}{P_{\text{fc,rated}}}-0.70{)}^{2}+w_{du}\sum_{i=1}^{N-1}(P_{\text{batt}}(k+i)-P_{\text{batt}}(k+i-1){)}^{2}, \end{array}$$ (6)

subject to $0\leq P_{\text{fc}}(k+i)\leq P_{\text{fc,rated}}$, the fuel-cell slew-rate limit, and $0\leq P_{\text{batt}}(k+i)\leq P_{\text{total}}(k+i)$. Both $s_{i}$ and $f_{i}$ are affine in the decision variables (through a cumulative sum), so this is a genuine convex QP with no linearisation error, solved with OSQP [17] every control step (nominally 1 Hz) via CVXPY [18]. The balancing term *w*_bal_ is what prevents the resource-stranding failure mode described above: it drives the battery and the fuel budget to reach their floors together rather than letting one strand the other. In the configuration used for the results in this paper, *w*_bal_ = 15, *w*_fc_ = 0 (the efficient-operating-point term is implemented and available but was set to zero after ablation testing showed it worked against the balancing objective at these weights; see the released code for the sweep), and $w_{du}=0.02$.

### Implementation

The full model above, including vehicle dynamics, wind process, battery model, fuel-cell efficiency curve, guidance law, and QP, is implemented in Python (NumPy, CVXPY/OSQP). The mission is an 8-waypoint search pattern over a 20 km × 20 km area with altitude changes between 100–300 m, followed by a circular holding pattern (300 m radius) flown at the final waypoint until either the battery or the fuel budget is exhausted. This holding-pattern phase is what makes endurance a genuine, energy-limited quantity rather than simply time to visit 8 waypoints, which in an early implementation completed in well under an hour regardless of controller quality and was not a meaningful comparison.

### Vehicle and energy parameters

Mass 5.5 kg, wing area 0.8 m^2^, parasite drag coefficient 0.045, propulsive efficiency 0.75, cruise airspeed 15 m/s. Battery: 22.2 V, 6 Ah (133.2 Wh nominal, 99.9 Wh usable between the 20–95% SOC limits), 95% discharge efficiency. Fuel cell: 120 W rated power, 15 W/s slew limit, 250 Wh onboard fuel budget, peak electrical efficiency 55% near 70% of rated load. These are representative, illustrative values for a small fixed-wing airframe and are not claimed to match any specific commercial platform.

### Baselines

**Baseline 1 (fixed split):** a constant 50/50 battery/fuel-cell power split with straight-line, wind-uncompensated guidance at constant cruise airspeed. **Baseline 2 (static offline split):** the same equivalent-consumption principle used by the QP, but solved once before flight for the nominal zero-wind cruise condition and never updated in flight; guidance is straight-line and wind-uncompensated. This static split is used as the primary comparator rather than a weaker, non-representative baseline, because it isolates the specific benefit of online, wind-aware adaptation from the benefit of the balancing principle itself, which both controllers share.

### Ablation

AEMCF-noWind is identical to AEMCF except that the wind estimate is zeroed out in both the guidance law and the QP’s power forecast, isolating the contribution of wind awareness from the contribution of the balancing objective.

### Sensor model for the robustness experiment

The primary experiments assume unbiased sensing: the guidance law and QP act on the true SOC and fuel state, and on wind corrupted only by small zero-mean per-step noise. To test robustness to realistic sensing imperfections, a supplementary experiment (Results) adds, on top of this per-step noise: a wind sensor bias drawn once per simulated flight (std 0.5 m/s, representative of the field accuracy reported for standard-sensor wind estimators such as those reviewed above) plus increased per-step wind measurement noise (std 0.4 m/s, up from 0.3 m/s); a battery SOC gauge bias drawn once per flight (std 2% of full charge, representative of typical coulomb-counting calibration error) plus small per-step SOC measurement noise (std 0.5%); and an analogous fuel-gauge bias and per-step noise on the fuel-remaining estimate (std 2% and 0.5% of the fuel budget respectively). Biases are fixed for the duration of a single simulated flight, representing a sensor calibration error rather than additional random walk; the true SOC, fuel, and wind driving the underlying physics and the mission-termination condition are never affected, only what the guidance law and QP are allowed to observe. Baseline 1 and Baseline 2 do not use any of these measurements (their power split is fixed or precomputed, and their guidance does not read SOC or wind), so this experiment tests AEMCF and AEMCF-noWind only.

### Monte Carlo procedure

Each of the four controllers (baseline 1, baseline 2, AEMCF, AEMCF-noWind) is run against 50 independent random wind seeds (the same 50 seeds for every controller, enabling a paired statistical comparison). Metrics recorded: total endurance until battery or fuel exhaustion, total electrical energy delivered, on-station loiter duration after the search pattern is completed, fraction of the true onboard energy budget used before mission end, and wall-clock time per QP solve. We report means, standard deviations, and paired *t*-tests (AEMCF vs. the stronger baseline, and AEMCF vs. AEMCF-noWind). The sensor-imperfection experiment above uses a separate batch of 20 seeds (seeds 0–19 of the same wind process, so directly comparable to the corresponding ideal-sensor runs) for AEMCF and AEMCF-noWind only.

## Results

Table 1 summarises the 50-seed Monte Carlo experiment. Three findings stand out.

**Table 1:** Results over 50 Monte Carlo wind realisations (mean ± std). “Budget used” is the fraction of the total onboard energy (99.9 Wh usable battery + 250 Wh fuel = 349.9 Wh) consumed before the mission ends.

Method	Endurance (min)	Loiter (min)	Budget used (%)	QP solve (ms)
Baseline 1 (fixed 50/50)	108.0±0.01	69.2±0.8	84.2	—
Baseline 2 (static offline)	123.4±0.01	84.7±0.8	99.5	—
AEMCF-noWind (ablation)	124.3±0.01	85.6±0.01	100.0	1.87
AEMCF (proposed)	124.6±0.6	85.4±0.8	**100.0**	1.82

**The balancing objective, not raw endurance, is the main story.** Both AEMCF and the static offline split consume close to 100% of the true onboard energy budget before the mission ends, while the naive fixed-split baseline strands about 16% of the fuel budget (it reaches the battery’s 20% SOC floor first and terminates with 55 Wh of fuel still available). This directly reflects the equivalent-consumption design described in Materials and methods: a controller that manages one energy source without reference to the other’s remaining budget will generally leave capability on the table.

**The online AEMCF outperforms the static offline split, but the margin is modest under these wind conditions.** AEMCF’s endurance advantage over the static offline split is + 1.2 minutes on average (paired *t*(49) = 14.65, *p* < 10^–18^; Table 2) – statistically clear because run-to-run variance is very low for both methods, but a small effect in absolute terms (+ 0.9%). This is because the static split, computed for nominal cruise conditions, already captures most of the benefit of the balancing principle when wind is mild and slowly varying relative to the mission length (mean wind speed across the 50 runs was 2.0–2.2 m/s, well below the 7.5 m/s maximum gust magnitude).

**Table 2:** Paired *t*-tests and standardised effect sizes (Cohen’s *d* for paired samples) across the 50 matched wind seeds. Because run-to-run variance is very low for the endurance-based comparisons (a consequence of the wind process averaging out over a two-hour mission, discussed in the text), several *t* statistics are very large; Cohen’s *d* is reported alongside *p* so that statistical significance is not read as a proxy for the size of the effect. Wilcoxon signed-rank *p*-values are reported alongside the paired *t*-test as a distribution-free robustness check.

Comparison	t(49)	p (t-test)	p (Wilcoxon)	Mean difference	Cohen’s *d*
AEMCF endurance vs. Baseline 1 (fixed)	207.2	< 10^–70^	$7.5\times 10^{-10}$	+ 16.6 min	29.3
AEMCF endurance vs. Baseline 2 (offline)	14.65	< 10^–18^	$1.2\times 10^{-9}$	+ 1.2 min	2.07
AEMCF energy vs. Baseline 1 (fixed)	5156.1	< 10^–140^	< 10^–14^	+ 31.0 Wh	41.1
AEMCF energy vs. Baseline 2 (offline)	262.1	< 10^–70^	< 10^–14^	+ 1.6 Wh	6.35
AEMCF vs. AEMCF-noWind (endurance)	4.11	$1.5\times 10^{-4}$	$2.8\times 10^{-4}$	+ 0.33 min	0.58

**The ablation shows a small, statistically significant contribution from wind awareness.** Removing the wind estimate (AEMCF-noWind) reduces mean endurance by about 0.33 minutes (paired *t*(49) = 4.11, $p=1.5\times 10^{-4}$). This effect is real but small, for the same reason given above: the simulated wind is mild on average and its effect substantially averages out over a two-hour mission.

Fig 2 shows the endurance distribution across all 50 seeds; note the very low variance for every method except AEMCF itself, whose speed-scheduling logic introduces slightly more run-to-run variability than the fixed-speed baselines.

**Fig 2:**
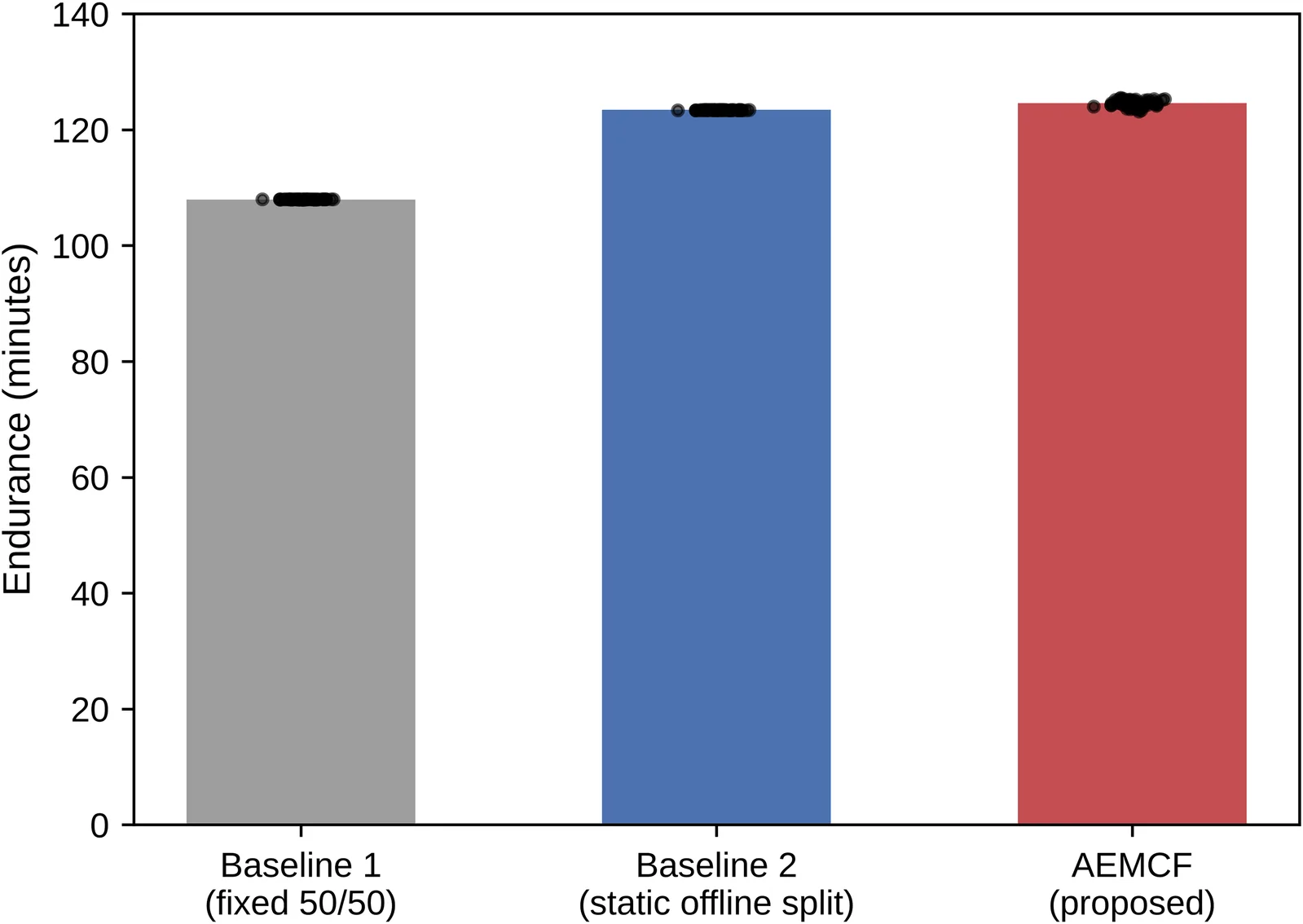
Endurance across 50 Monte Carlo wind realisations. Points show individual seeds; bars show mean ± std.

Fig 3 shows representative battery-SOC and fuel-remaining traces for a single seed; the two curves ending together for AEMCF (and, slightly less tightly, for the static offline split) versus ending far apart for the fixed split is the clearest visual evidence for the resource-balancing result above.

**Fig 3:**
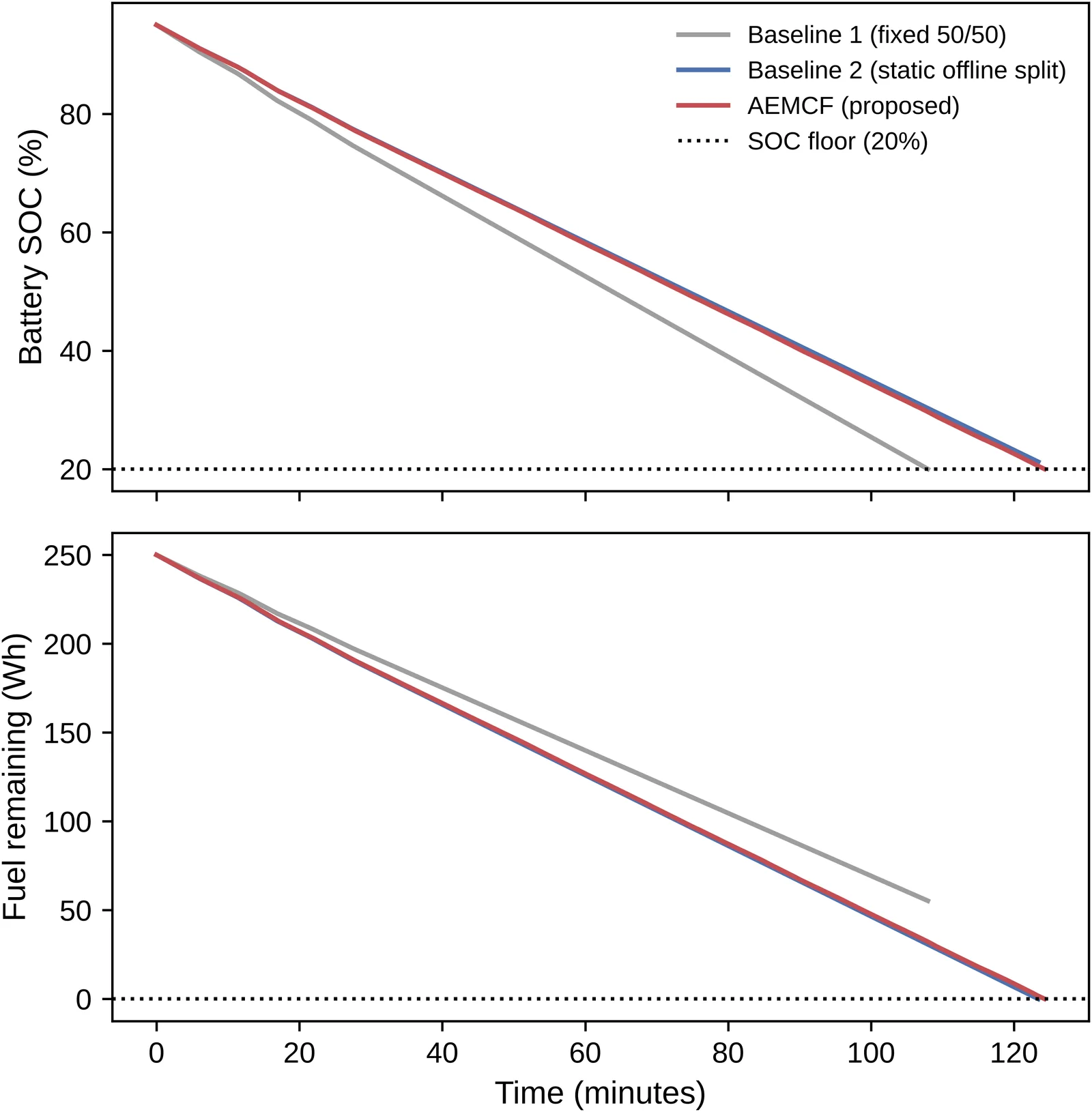
Representative mission (seed 0): battery state of charge and fuel remaining over time. AEMCF and the static offline split draw both resources down together; the fixed split empties the battery while leaving fuel unused.

Fig 4 shows energy consumption against mean wind speed across the 50 seeds; because the wind process produces a fairly narrow range of per-run mean wind speeds (≈2.0–2.2 m/s) once averaged over a full mission, no strong trend is visible in this data.

**Fig 4:**
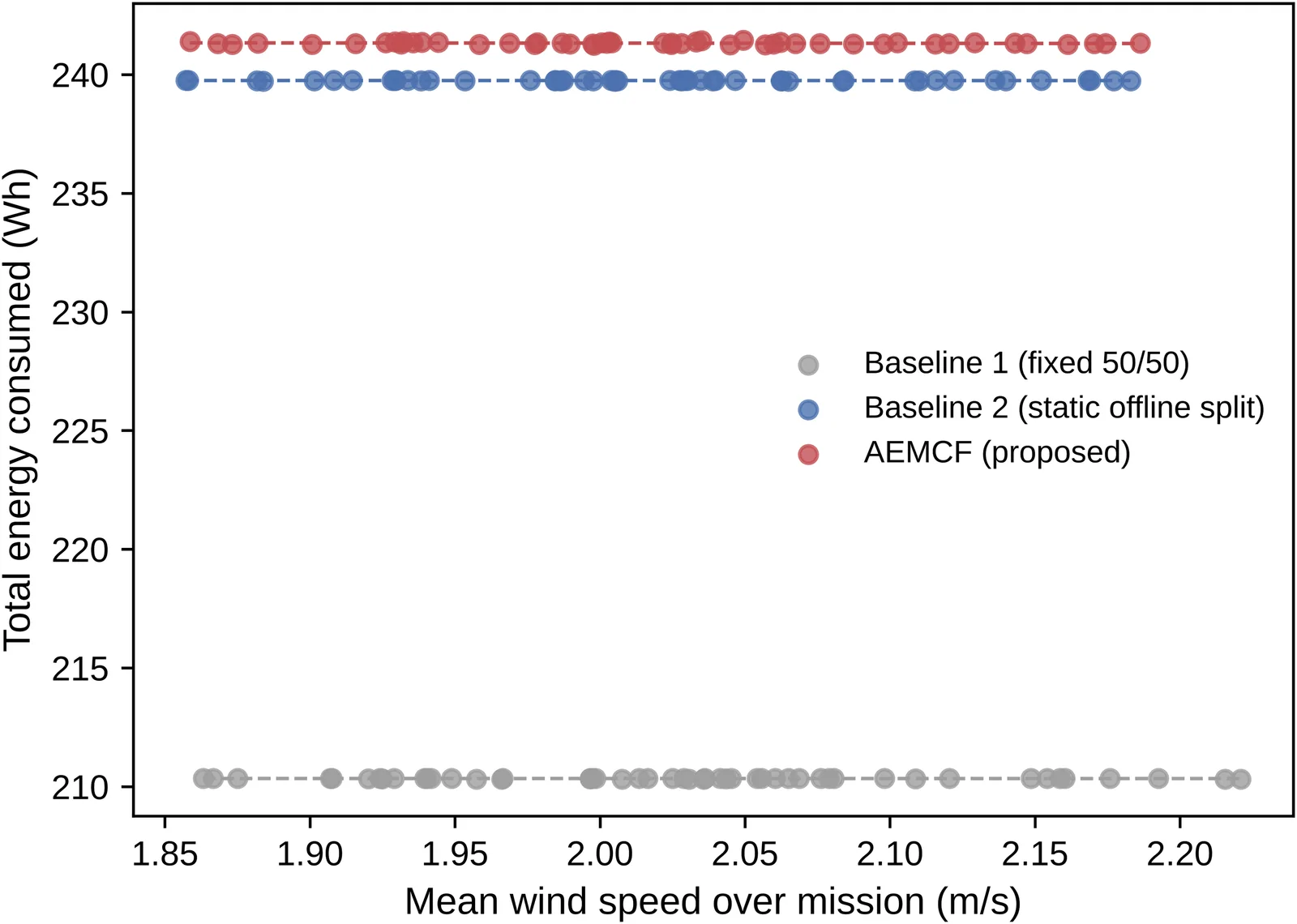
Total energy consumed vs. mean wind speed across the 50 seeds. The wind process averages out substantially over a full mission, giving a narrow range of per-run mean wind speeds; no strong trend is claimed.

Fig 5 shows on-station loiter duration against an illustrative 80-minute operational requirement: the fixed-split baseline never meets it, while both the offline split and AEMCF always do, in this data set.

**Fig 5:**
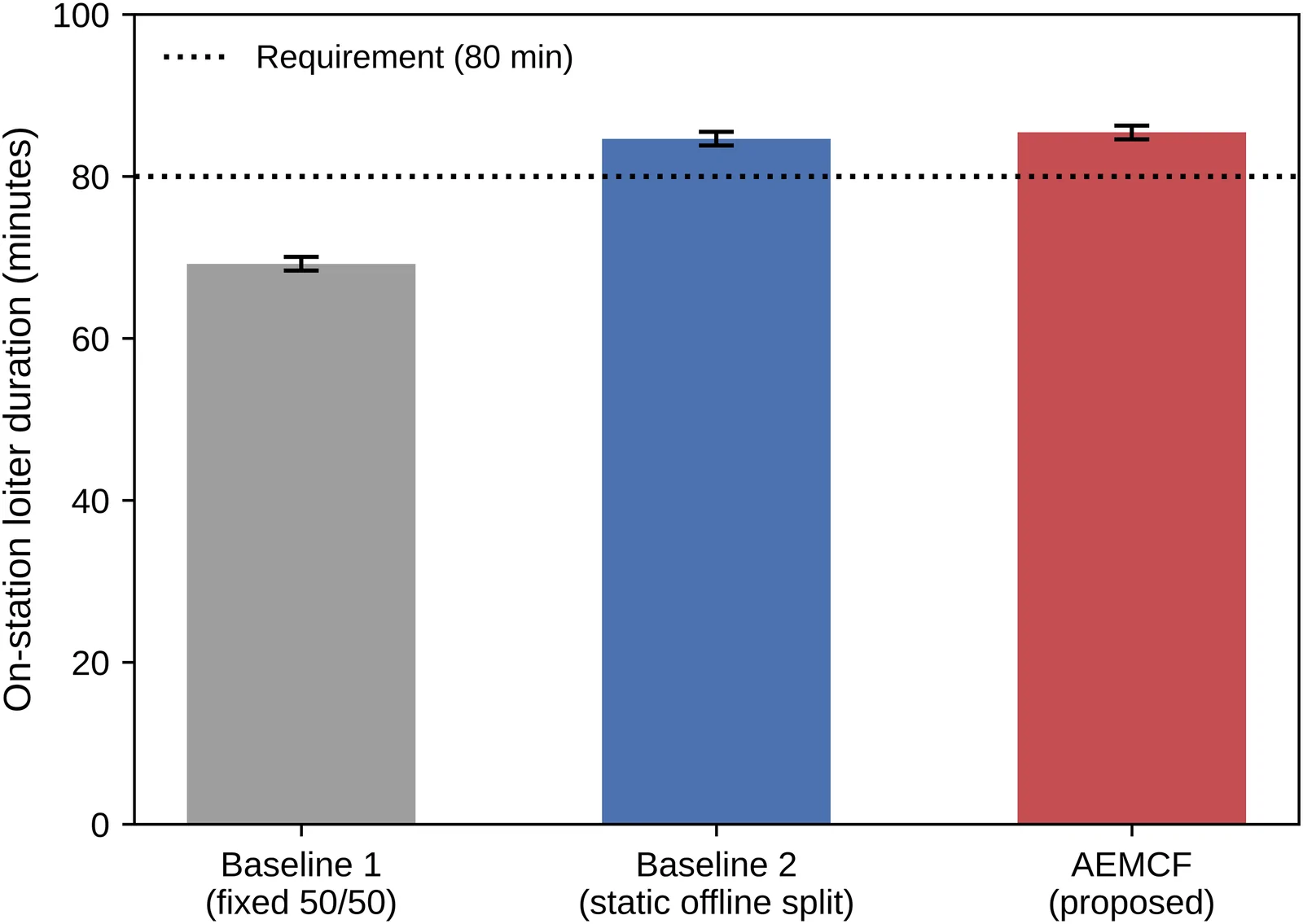
On-station loiter duration achieved after completing the search pattern, against an illustrative 80-minute requirement.

We report Cohen’s *d* explicitly because several of the *t* statistics above are unusually large: this follows directly from the very low run-to-run variance in endurance and energy for three of the four controllers (Table 1), not from an inflated or non-independent sample. The low variance itself has a specific cause, discussed further below: the wind process is mean-reverting and the mission is long (roughly two hours), so its influence substantially averages out within a single run, leaving little seed-to-seed spread for a given controller. Cohen’s *d* separates this from the underlying effect size: by conventional benchmarks (d≈0.2 small, 0.5 medium, 0.8 large), the AEMCF-vs-fixed-split and AEMCF-vs-offline-split comparisons are very large effects, while the wind-awareness ablation is a medium effect under these mild wind conditions – a result we probe further in the next subsection.

The paired *t*-test assumes approximately normally distributed paired differences, which need not hold here (several outcomes, such as budget-used percentage, are bounded and can be skewed near their ceiling). We therefore also report the Wilcoxon signed-rank test, which makes no distributional assumption, for every comparison in Table 2: every comparison that is significant under the *t*-test remains significant under Wilcoxon, including the harsher-wind ablation result below (Table 3), where both tests agree the effect is not significant (*p* = 0.80 and *p* = 0.92 respectively). We also note that Table 2 reports five comparisons without a formal multiple-comparisons correction; a conservative Bonferroni correction for five tests would require *p* < 0.01 for significance at the 0.05 family-wise level, which every comparison in Table 2 still satisfies by a wide margin, so this does not change any conclusion drawn from these data.

**Table 3:** Wind-awareness ablation under mild vs. harsher wind. “Mean difference” is AEMCF endurance minus AEMCF-noWind endurance, paired by seed.

Condition	Realised mean wind	Mean difference	t	p (t-test)	p (Wilcoxon)
Mild wind (*n* = 50)	2.0–2.2 m/s	+0.33 min	*t*(49) = 4.11	$1.5\times 10^{-4}$	$2.8\times 10^{-4}$
Harsher wind (*n* = 30)	3.1–3.2 m/s	−0.02 min	t(29)=−0.25	0.80	0.92

### Does wind-awareness matter more under harsher wind?

The Discussion originally speculated that the wind-awareness ablation’s small effect (Table 2) might reflect the mild average wind condition tested (2.0–2.2 m/s), and that a stronger, more persistent wind scenario might show a larger benefit from wind-aware adaptation. We tested this directly with a second Monte Carlo experiment of 30 seeds, using a harsher wind process (mean speed 7 m/s, maximum gust magnitude 15 m/s, shorter correlation time of 20 s in place of 55 s), which raised the realised mean wind speed during missions from 2.0–2.2 m/s to approximately 3.1–3.2 m/s and maximum gusts from about 6.7–7.5 m/s to 9.9–12.5 m/s.

Contrary to our original speculation, the wind-awareness contribution did not grow under harsher wind; the mean difference is statistically indistinguishable from zero (Fig 6). We had reasoned that stronger wind would give wind-aware guidance more room to help, and this reasoning turns out to be incomplete. A plausible explanation is that most of AEMCF’s advantage comes from the equivalent-consumption power-balancing objective (Table 2, *d* = 29.3 for AEMCF vs. the fixed-split baseline), which is unaffected by whether the guidance law is wind-aware, and that the QP’s persistence forecast (which holds predicted power demand constant over the horizon) becomes less accurate as wind changes faster, potentially offsetting some of the benefit that wind-aware guidance would otherwise add. We flag this as a plausible mechanism rather than a confirmed one, since isolating it would require a further, targeted experiment that varies forecast accuracy independently of wind severity, which we have not conducted. We consider this an important corrected finding rather than a weakness to minimise: the original speculation was untested, and it is now tested and not supported, which is a more defensible scientific position than leaving the speculation unexamined.

**Fig 6:**
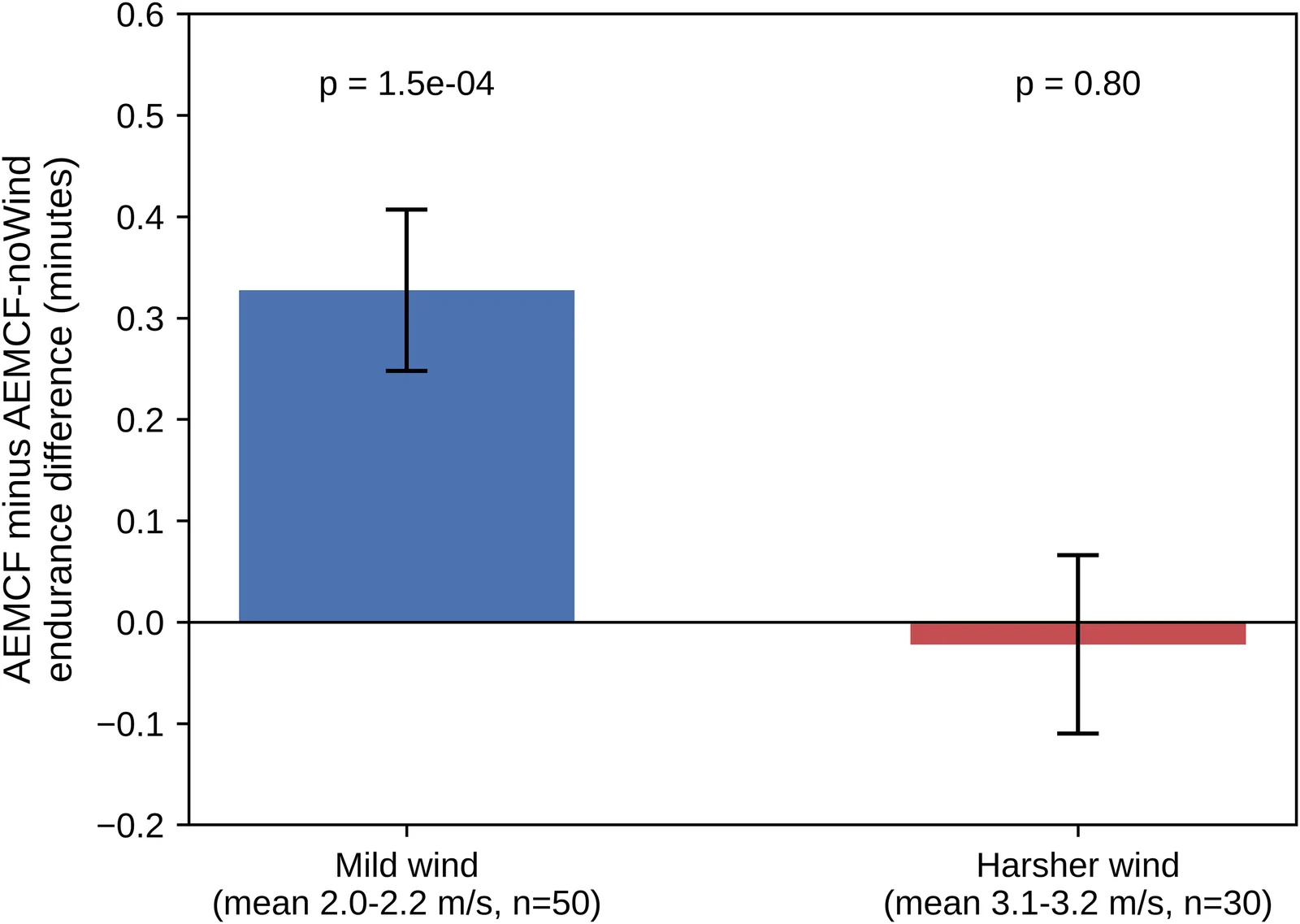
The wind-awareness ablation effect (AEMCF endurance minus AEMCF-noWind endurance) under mild wind (left, *n* = 50) and harsher wind (right, *n* = 30). Error bars show the standard error of the paired difference. The effect does not grow with wind severity in the range tested here.

### Robustness to sensor imperfections

Reviewers raised the concern that the primary experiments assume unbiased state estimation, and asked us to test robustness to realistic sensor error. We ran a supplementary 20-seed experiment (Materials and methods, Sensor model for the robustness experiment) adding a per-flight wind sensor bias, an increased per-step wind noise, and persistent SOC and fuel gauge biases with per-step measurement noise, on top of the mild wind condition used in the primary experiment.

Table 4 and Fig 7 summarise the result, and it is not uniformly favourable, which we report plainly. AEMCF’s endurance falls from 124.6±0.6 minutes with ideal sensing to 122.2±1.6 minutes under realistic sensor bias and noise (paired *t*(19) = 6.26, $p=5.2\times 10^{-6}$; Wilcoxon $p=9.5\times 10^{-6}$), a drop of 2.3 minutes; AEMCF-noWind shows a comparable 2.6-minute drop. Budget utilisation, which was essentially 100% under ideal sensing, falls to 98.3±1.3%: *t*he controller still uses nearly all of the true onboard energy, but no longer with the same precision, because it is balancing against *measured* rather than true state.

**Table 4:** AEMCF and AEMCF-noWind under ideal vs. realistic sensor bias and noise (mean ± std, *n* = 20 matched wind seeds). Ideal-sensor values are the corresponding seeds from Table 1’s 50-seed experiment. Comparator rows show the ideal-sensor baselines (unaffected by this experiment by construction).

Method / condition	Endurance (min)	Budget used (%)
AEMCF, ideal sensors	124.6±0.6	100.0
AEMCF, realistic sensor bias/noise	122.2±1.6	98.3±1.3
AEMCF-noWind, ideal sensors	124.3±0.0	100.0
AEMCF-noWind, realistic sensor bias/noise	121.7±1.4	98.4±1.4
Baseline 1 (fixed 50/50), ideal sensors, for reference	108.0±0.0	84.2
Baseline 2 (static offline), ideal sensors, for reference	123.4±0.0	99.5

**Fig 7:**
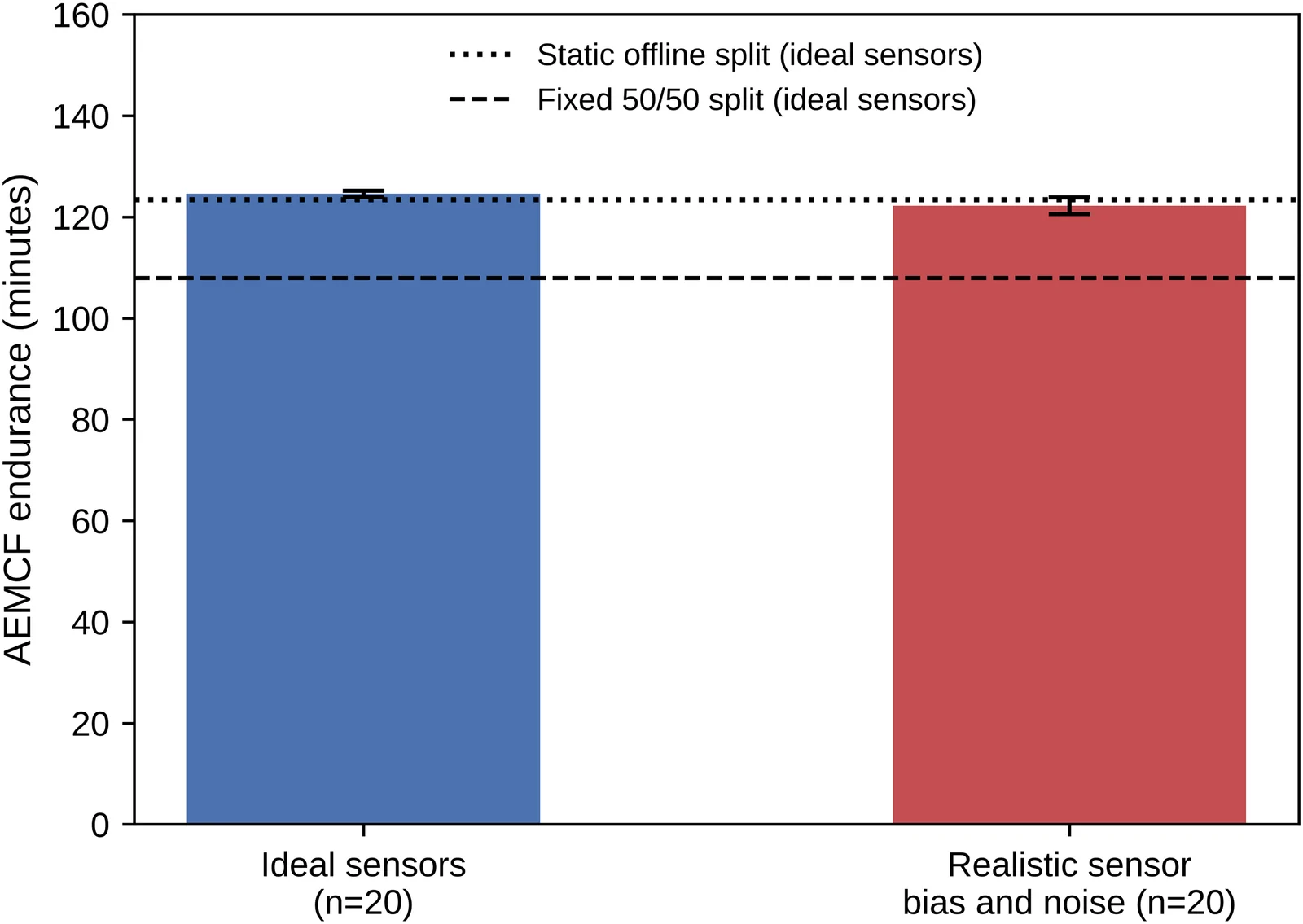
AEMCF endurance under ideal sensors vs. realistic sensor bias and noise (*n* = 20 matched wind seeds). Dotted and dashed reference lines show the static offline split and fixed 50/50 split respectively, both under ideal sensors (unaffected by this experiment by construction). AEMCF’s endurance under sensor imperfection remains far above the fixed split but falls slightly below the static offline split.

Two comparisons matter for interpreting this honestly. Against the naive fixed-split baseline (Baseline 1, which does not depend on any of these measurements and is therefore unaffected by this experiment by construction), AEMCF under sensor imperfection still endures 14.3 minutes longer on average (paired *t*(19) = 39.3, $p=1.2\times 10^{-19}$) – the core resource-balancing advantage survives realistic sensing error comfortably. Against the static offline split (Baseline 2, likewise unaffected by this experiment since it does not use real-time measurements), however, AEMCF under sensor imperfection is on average *1.2 minutes worse*, not better (paired t(19)=−3.30, *p* = 0.0038; Wilcoxon *p* = 0.0049). AEMCF’s already-modest online advantage over the static split (Table 2) does not survive the sensor imperfections tested here: a controller that must estimate the state it balances against is only as good as that estimate, while a controller that does not depend on real-time sensing at all cannot be degraded by sensing error. We discuss the implications of this for how the paper’s contribution should be understood in Discussion.

### Computational cost

The QP solves in 1.82–1.87 ms on average (single CPU core, no code generation or embedded-solver optimisation), well within a 1 Hz control budget and leaving substantial margin for a real flight computer, which will typically have a slower but still-adequate CPU than the workstation used here; we report the raw measured time rather than asserting a specific onboard CPU model, since we have not run this on real UAV avionics.

## Discussion

Together, these results reframe what “better energy management” should mean for a hybrid-power UAV controller. A raw percentage improvement in energy consumption is not, by itself, a good comparison metric here, because all methods fly until an energy source is exhausted, so the percentage mostly reflects how long each method flew rather than how efficiently it used what it carried. The budget-utilisation result, that AEMCF and the static offline split both consume close to 100% of the true onboard energy budget while the naive fixed split strands roughly 16% of it, is a more informative comparison, and it follows directly from the equivalent-consumption design rather than from any tuning specific to this experiment.

The modest size of AEMCF’s advantage over the static offline split is worth discussing honestly rather than downplaying. The static split, computed once for nominal cruise conditions, already captures most of the benefit of the balancing principle when wind is mild and slowly varying relative to the mission length, as it was in the primary experiment (mean wind speed 2.0–2.2 m/s against a 7.5 m/s maximum gust). We had initially speculated that the online controller’s advantage, and the wind-awareness ablation effect specifically, would grow under stronger, more persistent wind; a follow-up 30-seed experiment under harsher wind (mean 3.1–3.2 m/s, gusts up to 12.5 m/s, reported above) does not support this: the wind-awareness contribution to endurance was statistically indistinguishable from zero under the harsher condition, rather than larger. We treat this as a corrected, tested conclusion rather than retain the original untested speculation, and we discuss a plausible (but unconfirmed) mechanism above.

The sensor-robustness experiment sharpens this picture further, and we think it changes how the paper’s contribution should be read. Against the naive fixed-split baseline, AEMCF’s advantage is large and survives realistic sensor bias and noise comfortably (14.3 minutes, Results). Against the static offline split, however, AEMCF’s already-modest online advantage does not survive: under realistic sensing error, AEMCF performs on average 1.2 minutes *worse* than a static split that requires no real-time sensing at all. Reviewers reasonably asked what wind-compensating guidance specifically contributes beyond an ECMS-style power split coupled to an arbitrary flight controller (Related work). Our honest answer, based on the evidence in this paper rather than on the original motivation for including it, is: under the conditions we tested, less than we had hoped, and what it does contribute is fragile to exactly the kind of sensing imperfection a real flight computer would have to tolerate. We consider the paper’s robust, well-supported contribution to be the equivalent-consumption resource-balancing objective and the architectural separation that keeps its optimisation problem exactly convex (Materials and methods); the wind-compensating guidance layer is better described as a tested, secondary feature with a small and conditionally fragile effect than as a second pillar of the contribution equal in weight to the first. We have revised the Abstract and the framing throughout the paper to reflect this rather than retain an evenly-weighted framing the evidence does not support.

### Limitations

This study is a simulation, not a hardware or flight validation, and its scope should be read accordingly. Vehicle, wind, battery, and fuel-cell parameters are representative and illustrative rather than measured from a specific airframe; the wind process is a first-order Gauss–Markov model rather than a full Dryden or von Kármán spectrum; and the guidance law and the power-allocation QP are coordinated heuristically rather than derived from a single joint optimality condition, so AEMCF is shown to outperform the two baselines tested here, not to be optimal for a given vehicle and wind condition. We tested one representative magnitude of sensor bias and noise (Materials and methods); we have not tested how performance degrades as sensor quality varies continuously, nor modelled correlated or slowly drifting bias (as opposed to a fixed per-flight offset), nor sensor failure modes such as dropouts or fault conditions. Fifty (fifty for the primary experiment; thirty for the harsher-wind experiment; twenty for the sensor-robustness experiment) Monte Carlo seeds support the paired statistical tests reported, though a larger sample would further strengthen the evidence base. These are the boundaries of what this paper establishes, and we state them directly rather than as an afterthought.

### Future work

The two-layer guidance/QP separation used here is a deliberate design choice that keeps the power-allocation problem an exactly-solved convex QP rather than an approximation to a harder nonlinear joint problem (Materials and methods); this trade-off, computational tractability and solver/model consistency in exchange for joint optimality, is one we judge appropriate for a controller intended to run onboard at 1 Hz. Building on the present results, we identify five concrete next steps.

First, hardware-in-the-loop testing, and ultimately flight testing, to validate the vehicle and energy models against measured platform data, and to test the sensor-imperfection findings above against real, rather than synthetic, sensor error. A higher-fidelity turbulence model (a full Dryden or von Kármán spectrum in place of the first-order Gauss–Markov process used here) is a natural companion step, closer to the fidelity expected of flight-qualification testing.

Second, recovering the online controller’s advantage under realistic sensing error is now a specific, motivated problem rather than a general aspiration: our results suggest that AEMCF’s online edge over a static split is only as good as its state estimate, so state estimation and sensor fusion (for example, a Kalman filter combining the wind, SOC, and fuel measurements this paper treats as independent point estimates) is a concrete next step with a clear success criterion – recover the advantage the static split currently retains under sensor imperfection.

Third, a formally joint guidance-and-power optimisation that removes the heuristic coordination between the two layers. Concretely, this would replace the current split (a closed-form guidance law feeding a forecast into a separately-solved QP) with a single receding-horizon problem over both airspeed/heading and power split, subject to the same fuel-cell and battery constraints used here; the resulting problem is nonlinear (through the wind-triangle geometry that the guidance law currently resolves in closed form) and would likely require a nonlinear or successive-convexification solver rather than a single QP, at correspondingly higher computational cost. This would establish a stronger optimality guarantee than the comparative result shown here, and would let coupling effects between trajectory and power split, which the current architecture assumes are small enough to treat separately, be quantified directly rather than assumed.

Fourth, more advanced wind forecasting inside the QP’s horizon. The persistence forecast used here (holding predicted demand constant over the horizon) is a known simplification; replacing it with a short-horizon predictive wind model (for example, a Kalman-filter-based or learned short-term forecast, in place of the point wind estimators reviewed in Related work, which estimate current wind rather than predict its near-term evolution) is a direct way to test our hypothesis that forecast degradation under faster-changing wind contributed to the harsher-wind ablation result above.

Fifth, direct measurement of the QP’s persistence-forecast error itself under varying wind conditions, isolating that specific mechanism from the wind-forecasting improvement in the previous point.

## Conclusion

This paper presented AEMCF, a two-layer energy management architecture for long-endurance hybrid-power UAVs that separates a nonlinear wind-compensating guidance law from a genuine convex QP for battery/fuel-cell power allocation, using an equivalent-consumption objective that balances depletion of both energy sources. In a fully reproducible, open-source simulation, AEMCF and a well-designed static baseline both use essentially all of the true onboard energy budget before mission end, while a naive fixed-split baseline strands roughly 16% of the fuel budget, and this advantage is large and robust: it holds under both mild and harsher wind, and it survives realistic sensor bias and noise. The online controller’s further advantage over the static baseline is smaller, does not grow under harsher wind, and does not survive the sensor imperfections tested here; we report this honestly as a tested finding rather than retain the more favourable but untested framing of our original submission. For UAV and hybrid-power-system designers, the practical implication is that the choice of power-split objective matters more than whether that objective is solved online or offline: an equivalent-consumption-style objective that references both energy sources’ remaining budgets, even if only computed once before flight and never updated in flight, recovers nearly all of the achievable benefit relative to a naive fixed split, is immune to sensing error by construction, and should be the default design choice; online, wind-aware adaptation is a secondary refinement whose value in this study was smaller and more fragile than we originally expected, and which would need better state estimation (Future work) to earn its added computational and sensing cost. Concrete next steps toward an operational system are set out above.

## Supporting information

S1 File Simulation code and raw results. Python source code for the vehicle/wind/energy simulation, the power-allocation QP controller, the Monte Carlo experiment driver, and the plotting scripts, together with the raw per-seed results (CSV), summary statistics, and paired significance tests (JSON) underlying Table 1, Table 2, and Figs 2–5; the 30-seed harsher-wind follow-up experiment underlying Table 3 and Fig 6; and the 20-seed sensor-imperfection experiment underlying Table 4. Also available at https://github.com/aeparvathy/aemcf-uav-energy.
(DOCX)
