# Supplementary material for: An adaptive energy management and control framework for long-endurance UAV missions in dynamic environments: A reproducible simulation study
Source: PLoS One. 2026 Nov 5;21(11):e0361428. doi: 10.1371/journal.pone.0361428 (PMC13739226; doi:10.1371/journal.pone.0361428)
Supplement: Supplementary file 1 [file pone.0361428.s003.pdf]

Response to Reviewer Comments — Second Round

Manuscript PONE-D-26-43977: "An adaptive energy management and control framework for long-endurance UAV missions in dynamic environments: a reproducible simulation study"

We thank both reviewers and the Academic Editor for a constructive second review. Both reviewers rated the manuscript "Yes" on technical soundness, statistical rigour, data availability, and clarity, and both offered suggestions for further strengthening the work rather than objections to publication. We have used every substantive point to add new material to the manuscript, including a new experiment we designed specifically in response to these comments. This document addresses each point in turn; reviewer comments are reproduced in italics.

Reviewer #1

General assessment

Reviewer #1 wrote: *"This work proposes AEMCF... In all, this work seems well prepared."*

We are grateful for this assessment and address the three suggestions below.

Point 1.1: Integrate more advanced wind-driven predictive forecasting techniques

Reviewer comment: *"Suggest integrate more advanced wind-driven predictive forecasting techniques."*

Response: We agree and have not implemented this in the present revision, since it is a substantial undertaking in its own right, but we have added it as an explicit, concrete item in a new Future Work section (fourth of five items): replacing the QP's current persistence forecast (which holds predicted power demand constant over the horizon) with a short-horizon predictive wind model, for example Kalman-filter-based or learned short-term forecasting, as distinct from the point wind estimators already reviewed in Related Work, which estimate current wind rather than predict its near-term evolution. We tie this directly to a finding elsewhere in this revision: our new harsher-wind experiment (added in response to the first round of review) found that the wind-awareness benefit did not grow as expected under stronger wind, and we hypothesise that persistence-forecast degradation at faster wind time

scales is a contributing mechanism. Better forecasting is therefore not just a general improvement but a direct test of that hypothesis.

Point 1.2: Consider sensor imperfections — bias, wind estimation drift, SOC measurement error

Reviewer comment: *"Suggest consider sensor imperfections. Introduce sensor bias, wind estimation drift and SOC measurement error to test real-world robustness."*

Response: We have done this as a new experiment, not only as a discussion addition. We added configurable, persistent per-flight sensor bias plus per-step measurement noise to the wind estimate, the battery SOC reading, and the fuel-remaining reading (new Materials and methods subsection, "Sensor model for the robustness experiment"), at magnitudes representative of typical wind-estimator field accuracy and typical coulomb-counting/flow-integration gauge calibration error. We ran a new 20-seed Monte Carlo experiment under this sensor model (new Results subsection, "Robustness to sensor imperfections", new Table 4, new Fig 7).

The result is genuinely mixed, and we report it exactly as found rather than selectively. AEMCF's advantage over the naive fixed-split baseline survives sensor imperfection comfortably (14.3 minutes). Its smaller advantage over the static offline split does not: under realistic sensor bias and noise, AEMCF performs on average 1.2 minutes worse than the static split (paired $t(19)=-3.30$, $p=0.0038$). We discuss the implications of this directly in Discussion and use it to reframe which part of AEMCF's contribution we consider well supported by the evidence (the resource-balancing objective) versus more fragile (the wind-compensating guidance layer). We consider this a more rigorous and more honest response than adding sensor noise only as a caveat without testing its effect.

Point 1.3: Develop a joint optimisation formulation for guidance and power allocation

Reviewer comment: *"Guidance and power allocation are solved separately without unified optimality conditions. Suggest develop a joint optimisation formulation to capture full coupling between flight trajectory and power split."*

Response: We agree this is the correct next step and have not attempted the full joint formulation in this revision, since it changes the optimisation problem's structure (from a convex QP to a nonlinear or successive-convexification problem, discussed below) and is a substantial piece of work in its own right. We have added a concrete sketch of the formulation

in the new Future Work section (third of five items): a single receding-horizon problem over both airspeed/heading and power split, subject to the same fuel-cell and battery constraints used here, in which the wind-triangle geometry that the guidance law currently resolves in closed form would instead enter the optimisation directly, making the problem nonlinear and requiring a nonlinear or successive-convexification solver rather than a single QP, at correspondingly higher computational cost. We also now state explicitly in the new Limitations section that AEMCF is shown to outperform the two baselines tested, not proven optimal for a given vehicle and wind condition, which follows directly from this gap.

Reviewer #2

Point 2.1: Distinguish AEMCF from standard ECMS and A-ECMS

Reviewer comment: *"The authors should explicitly distinguish AEMCF from standard ECMS and its adaptive variants (A-ECMS). What does 'wind-compensating guidance' add that a standard ECMS coupled with any flight controller cannot achieve? Without this contrast, the novelty appears incremental rather than substantive."*

Response: This is a fair challenge and we have added a substantial new paragraph in Related Work addressing it directly with two specific, testable distinctions rather than a general claim of novelty. First, the balancing term is not a fuel-cost equivalence factor at all: rather than converting battery energy into an equivalent fuel price (a quantity A-ECMS must estimate or adapt online from drive-cycle recognition or a running trip-energy estimate), it equates the fractional depletion of the battery and fuel budgets directly, using only the two capacities as fixed, known design constants. This removes the equivalence-factor calibration problem central to the ECMS/A-ECMS literature. Second, the guidance law's airspeed scheduling reads the same battery state of charge that the power-allocation QP tracks, so the vehicle's own power demand is adjusted with reference to the same energy state the power split is protecting; a generic flight controller paired with ECMS would fly a nominal airspeed profile with no knowledge of the battery or fuel state. We explicitly frame whether this coupling matters as an empirical question rather than assert it, and answer it honestly using the evidence in this paper (see Point 2.2 below): the effect is real but small under the conditions tested.

Point 2.2: Strengthen or justify the wind-guidance contribution

Reviewer comment: *“The authors should either strengthen the wind-guidance contribution or justify its inclusion with a clearer mechanism.”*

Response: We have chosen honest justification over overstatement, because that is what the evidence in this paper, including the new sensor-robustness experiment described under Point 1.2, supports. We have revised the Discussion, Abstract, and Conclusion throughout this manuscript to reframe the paper's contribution accordingly: the equivalent-consumption resource-balancing objective is the paper's robust, well-supported contribution (large advantage over the naive baseline, holding under both harsher wind and realistic sensor imperfection), while the wind-compensating guidance layer is now described as a tested, secondary feature with a small effect that does not grow under harsher wind and does not survive realistic sensor noise, rather than as a second pillar of the contribution equal in weight to the first. We believe this reframing directly answers both this point and Point 2.1: the paper's novelty rests principally on the balancing formulation and the architectural separation that keeps its optimisation exactly convex, and we no longer claim more for the wind-compensating layer than our own results support.

Point 2.3: Discuss the validity gap between simulation and flight, or validate against higher-fidelity models

Reviewer comment: *“The authors should discuss the validity gap between this simulation and actual flight conditions, or provide validation against higher-fidelity models.”*

Response: We have both discussed this gap explicitly and taken one concrete step to narrow it. The new Limitations section states plainly that this is a simulation study, not a hardware or flight validation, and specifies exactly what has and has not been tested (one representative magnitude of sensor bias/noise; no continuous variation in sensor quality; no correlated or slowly drifting bias; no sensor failure modes). The new Future Work section's first item names hardware-in-the-loop and flight testing as the step that would close this gap, together with adopting a full Dryden or von Kármán turbulence spectrum in place of the first-order Gauss–Markov process used throughout this paper, which we agree is closer to the fidelity expected of flight-qualification testing. The concrete step we have taken within this revision is the sensor-imperfection experiment described under Point 1.2, which is a direct, if partial, test of how the validity gap affects AEMCF's specific claims rather than only a discussion of the gap in the abstract.

Point 2.4: Additional citations

Reviewer comment: *"More related references should be added in this paper, for example, Joint optimisation of quality-dependent production and economic-dependent maintenance… [five references on production/maintenance optimisation and reliability engineering]."*

Response:

We thank the reviewer for these suggestions. Four of the five [16,17,18,19] are now cited in Related Work, alongside a sentence noting that they address joint optimisation of two coupled operational objectives in production and maintenance scheduling rather than energy management, which is the connection relevant to this paper's own two-objective balancing design and is great addition for general reader to have a understand the joint optimisation.

Summary of additions made to the manuscript in this round

1. New sensor-imperfection experiment (20 seeds): new Materials and methods subsection, new Results subsection, new Table 4, new Fig 7, directly responding to Reviewer #1 Point 1.2 and contributing to Reviewer #2 Point 2.3.
2. New Related Work paragraph explicitly distinguishing AEMCF from ECMS/A-ECMS with two specific technical distinctions, responding to Reviewer #2 Point 2.1.
3. Abstract, Discussion, and Conclusion revised throughout to honestly reframe the paper's principal contribution as the resource-balancing objective, with wind-compensating guidance described as a secondary, tested, conditionally fragile feature, responding to Reviewer #2 Point 2.2.
4. Future Work expanded from four to five items, adding a concrete joint-optimisation formulation sketch (Reviewer #1 Point 1.3), advanced wind forecasting (Reviewer #1 Point 1.1), a state-estimation/sensor-fusion proposal motivated directly by the new sensor experiment, and higher-fidelity turbulence modelling and flight validation (Reviewer #2 Point 2.3).
5. 4 new references added.
6. All code and data, including both new experiments, are available at https://github.com/aeparvathy/aemcf-uav-energy.

We are grateful to both reviewers for comments that led us to test claims we had previously only asserted or hoped for, and we believe the revised manuscript is more honest about the size and robustness of its contribution as a result.

Parvathy Rajendran (corresponding author)

On behalf of: Jiazheng Wang, Parvathy Rajendran, Aohua Li, Shirui Wang

1. Wang J, Luo L, Song D, Tang X. Joint optimisation of quality-dependent production and economic-dependent maintenance for a degradation system under online and offline inspections. Int J Prod Res. 2026. doi:10.1080/00207543.2026.2718795.
2. Wang J, Luo L, Mu G, Ma Y, Ni C. Joint optimization of quality control and maintenance policy for a production system with quality-dependent failures. Expert Syst Appl. 2025;272:126800. doi:10.1016/j.eswa.2025.126800.
3. Ma Y, Wei F, Kang Z, Qiu Q, Ma X, Yang L. Multi-level adaptive risk control: synergistic maintenance-termination optimization with online parameter learning. Reliab Eng Syst Saf. 2026;270:112138. doi:10.1016/j.ress.2025.112138.
4. Yang A, Qiu Q, Zhu M, Cui L, Chen W, Chen J. Condition-based maintenance strategy for redundant systems with arbitrary structures using improved reinforcement learning. Reliab Eng Syst Saf. 2022;225:108643. doi:10.1016/j.ress.2022.108643.
