# Supplementary material for: An adaptive energy management and control framework for long-endurance UAV missions in dynamic environments: A reproducible simulation study
Source: PLoS One. 2026 Nov 5;21(11):e0361428. doi: 10.1371/journal.pone.0361428 (PMC13739226; doi:10.1371/journal.pone.0361428)
Supplement: Supplementary file 2 [file pone.0361428.s001.docx]

\section*{Supporting information}

\paragraph*{S1 File.} \label{S1_File}

\textbf{Simulation code and raw results.} Python source code for the vehicle/wind/energy simulation, the power-allocation QP controller, the Monte Carlo experiment driver, and the plotting scripts, together with the raw per-seed results (CSV), summary statistics, and paired significance tests (JSON) underlying Table~\ref{table1}, Table~\ref{table2}, and Figs~\ref{fig2}--\ref{fig5}; the 30-seed harsher-wind follow-up experiment underlying Table~\ref{table3} and Fig~\ref{fig6}; and the 20-seed sensor-imperfection experiment underlying Table~\ref{table4}. Also available at \url{https://github.com/aeparvathy/aemcf-uav-energy}.
