# Supplementary material for: An adaptive energy management and control framework for long-endurance UAV missions in dynamic environments: A reproducible simulation study
Source: PLoS One. 2026 Nov 5;21(11):e0361428. doi: 10.1371/journal.pone.0361428 (PMC13739226; doi:10.1371/journal.pone.0361428)
Supplement: Supplementary file 3 [file pone.0361428.s002.docx]

**Response to Reviewer Comments**

*Manuscript PONE-D-26-39301: “An adaptive energy management and control framework for long-endurance UAV missions in dynamic environments: a reproducible simulation study”*

We thank Reviewer #1 for a careful and substantive review. The manuscript was not accepted on this occasion, but every point raised was specific and actionable, and we have used them to materially revise the work rather than only the text. This document responds to each point in turn and describes exactly what changed as a result. Reviewer comments are reproduced in italics; our response follows each one.

**General assessment**

Reviewer #1 wrote: *“This work develops AEMCF, a two-layer adaptive framework for fuel-cell-battery hybrid long-endurance UAVs. It separates nonlinear wind-compensating guidance from a receding-horizon convex quadratic-program power allocator built on equivalent-consumption principles, avoiding solver-model mismatch. Though it has done some works, I do not recommend acceptance.”*

**Point 1: Wind is mild (2.0–2.2 m/s) and the wind-awareness endurance gain is small (0.33 min)**

Reviewer comment: *“Simulated wind has low average speed (2.0–2.2 m/s), only mild gusts. The endurance gain from online wind-aware adaptation is merely 0.33 min.”*

**Response:** This was already disclosed as a limitation in the submitted manuscript, but we agree that a stated limitation is not a substitute for testing it. We have now run a second Monte Carlo experiment (30 seeds) under a substantially harsher wind process: mean speed raised from 4 m/s to 7 m/s and maximum gust magnitude from 7.5 m/s to 15 m/s, with a shorter correlation time (20 s vs. 55 s) to make gusts more frequent, together raising the realised in-mission mean wind speed from 2.0–2.2 m/s to approximately 3.1–3.2 m/s and maximum gusts from 6.7–7.5 m/s to 9.9–12.5 m/s.

The result is not what we had hypothesised, and we report it exactly as found: the wind-awareness endurance contribution did not grow under harsher wind. It fell from a statistically significant +0.33 minutes (t(49)=4.11, p=1.5×10⁻⁴, mild wind) to a statistically indistinguishable-from-zero −0.02 minutes (t(29)=−0.25, p=0.80, harsher wind). We have added this as a new Results subsection (“Does wind-awareness matter more under harsher wind?”), a new table and figure, and a revised Discussion paragraph that replaces our original untested speculation with this tested finding, together with a plausible (explicitly labelled as unconfirmed) mechanistic explanation: most of AEMCF's advantage comes from the power-balancing objective rather than from wind-aware guidance specifically, and the QP's persistence forecast may become less accurate as wind changes faster, which could offset part of the benefit wind-aware guidance would otherwise add.

**Point 2: No hardware-in-the-loop or flight testing; simplified, uncalibrated models; sensor bias and estimation error omitted**

Reviewer comment: *“No hardware-in-the-loop or real flight tests. Vehicle, fuel-cell and wind models are simplified illustrative parametrizations instead of real-platform calibrated data. Sensor bias and state-estimation errors are omitted, so real-world robustness is unknown.”*

**Response:** This is correct, and we do not think it can be fully resolved within a simulation-only study. We have restructured the end of the manuscript into a separate Limitations section (stating plainly what this study does and does not establish) and a new Future Work section, which lists hardware-in-the-loop testing, and ultimately flight testing, as the first of four concrete next steps, described as necessary rather than merely desirable before any operational claim could be made. We have not fabricated or implied platform-calibrated data anywhere in the manuscript, and all vehicle, battery, and fuel-cell parameters are explicitly labelled as representative and illustrative rather than measured. We consider this an honest scope boundary of the present contribution (a controller design and a reproducible simulation methodology) rather than a defect that additional simulation work could paper over, and we have said so directly in the revised text.

**Point 3: Guidance and power allocation are not derived from a unified optimality formulation; coupling effects are ignored**

Reviewer comment: *“Guidance law and power allocation are not derived from a unified optimality formulation. Potential coupling effects are ignored, leaving theoretical optimality unestablished.”*

**Response:** This is correct as stated, and we have expanded our discussion of it to explain the reasoning rather than only name the gap. The two-layer separation (a closed-form nonlinear guidance law and a separately-solved convex QP) is a deliberate design choice: it is precisely what keeps the power-allocation problem an exactly-solved convex quadratic program, rather than an approximation to a harder nonlinear joint problem, and this is what avoids the solver/model inconsistency we identify as a common issue in this literature. Our new Limitations section now states explicitly that this trade-off, computational tractability and solver/model consistency in exchange for forfeiting joint optimality, is a real limitation of the theoretical guarantees AEMCF can claim: we can show that it outperforms the two tested baselines, not that it is optimal for a given vehicle and wind condition. A formally joint guidance-and-power optimisation is identified in the new Future Work section as a specific, valuable next step that this paper does not attempt.

**Point 4: Statistical analysis performed appropriately and rigorously? (Reviewer scored: No)**

Reviewer comment: *This question was scored “No” without an accompanying prose comment. We have nonetheless treated it as a substantive point and strengthened the statistical presentation on our own initiative, since we could not ask the reviewer to clarify which aspect was of concern.*

**Response:** We identified two concrete gaps and addressed both directly. First, several of our paired t-test statistics are very large (t up to several thousand), which can look suspicious without context; we added Cohen's d effect sizes alongside every t-test so that statistical significance is not mistaken for effect magnitude, and explained that the large t-statistics follow from genuinely low run-to-run variance (itself explained by the wind process averaging out over a two-hour mission), not from an inflated or non-independent sample. Second, the paired t-test assumes approximately normal paired differences, which need not hold for bounded outcomes such as budget-used percentage; we added the Wilcoxon signed-rank test, which makes no distributional assumption, alongside every t-test as a robustness check. Every comparison that is significant under the t-test remains significant under Wilcoxon, and the harsher-wind null result (Point 1, above) is null under both tests. We also explicitly addressed multiple comparisons: five tests are reported without correction, but a conservative Bonferroni threshold (p<0.01 for five tests at family-wise α=0.05) is still cleared by every comparison, so this does not change any conclusion. If the reviewer's concern was a different aspect of the statistical treatment, we welcome that clarification and will address it directly.

**Summary of manuscript changes**

• New 30-seed harsher-wind Monte Carlo experiment, with a new Results subsection, table, and figure, directly testing (and correcting) our original speculation about wind severity; this finding is now also reflected in a revised abstract sentence.

• Cohen's d effect sizes and Wilcoxon signed-rank tests added alongside every paired t-test, to separate statistical significance from effect magnitude and to check the t-test's normality assumption; a multiple-comparisons check (Bonferroni) is also reported explicitly.

• The end of the manuscript is now split into a dedicated Limitations section (stating plainly, without hedging, what this simulation study does and does not establish) and a separate Future Work section (four concrete next steps: hardware-in-the-loop and flight testing; modelling sensor bias and estimation error; a formally joint guidance-and-power optimisation; and direct measurement of QP forecast error under varying wind, motivated by the harsher-wind finding above).

• All changes, code, and raw data (including the new harsher-wind experiment) are available at https://github.com/aeparvathy/aemcf-uav-energy for independent verification.

We are grateful to Reviewer #1 for comments that improved the manuscript's honesty and precision, and we believe the revised version represents a materially stronger and more carefully bounded contribution.

Parvathy Rajendran (corresponding author)

*On behalf of: Jiazheng Wang, Parvathy Rajendran, Aohua Li, Shirui Wang*
