# Supplementary material for: An adaptive energy management and control framework for long-endurance UAV missions in dynamic environments: A reproducible simulation study
Source: PLoS One. 2026 Nov 5;21(11):e0361428. doi: 10.1371/journal.pone.0361428 (PMC13739226; doi:10.1371/journal.pone.0361428)
Supplement: Peer review history

# Author comment: Author response to Decision Letter 0

**DOI:** 10.1371/journal.pone.0361428.r001

7 Sep 2026

Attachment Submitted filename: Response_to_Reviewers.docx

# Peer review: Decision Letter 0

**DOI:** 10.1371/journal.pone.0361428.r002

**Authors:** Qingan Qiu

21 Sep 2026

Dear Dr. Rajendran,

Thank you for submitting your manuscript to PLOS One. After careful consideration, we feel that it has merit but does not fully meet PLOS One’s publication criteria as it currently stands. Therefore, we invite you to submit a revised version of the manuscript that addresses the points raised during the review process.

**ACADEMIC EDITOR: Two reviewers provide very important comments for further improvement. Please address the comments with detailed response.**

Please submit your revised manuscript by Nov 20 2026 11:59PM. If you will need more time than this to complete your revisions, please reply to this message or contact the journal office at plosone@plos.org. When you're ready to submit your revision, log on to https://www.editorialmanager.com/pone/ and select the 'Submissions Needing Revision' folder to locate your manuscript file.

- A letter that responds to each point raised by the academic editor and reviewer(s). You should upload this letter as a separate file labeled 'Response to Reviewers'.
- A marked-up copy of your manuscript that highlights changes made to the original version. You should upload this as a separate file labeled 'Revised Manuscript with Track Changes'.
- An unmarked version of your revised paper without tracked changes. You should upload this as a separate file labeled 'Manuscript'.

If you would like to make changes to your financial disclosure, please include your updated statement in your cover letter. Guidelines for resubmitting your figure files are available below the reviewer comments at the end of this letter.

If applicable, we recommend that you deposit your laboratory protocols in protocols.io to enhance the reproducibility of your results. Protocols.io assigns your protocol its own identifier (DOI) so that it can be cited independently in the future. For instructions see: https://journals.plos.org/plosone/s/submission-guidelines#loc-laboratory-protocols. Additionally, PLOS One offers an option for publishing peer-reviewed Lab Protocol articles, which describe protocols hosted on protocols.io. Read more information on sharing protocols at https://plos.org/protocols?utm_medium=editorial-email&utm_source=authorletters&utm_campaign=protocols.

As the corresponding author, your ORCID iD is verified in the submission system and will appear in the published article. PLOS supports the use of ORCID, and we encourage all coauthors to register for an ORCID iD and use it as well. Please encourage your coauthors to verify their ORCID iD within the submission system before final acceptance, as unverified ORCID iDs will not appear in the published article. *Only*  the individual author can complete the verification step; PLOS staff *cannot*  verify ORCID iDs on behalf of authors.

We look forward to receiving your revised manuscript.

Kind regards,

Qingan Qiu

Academic Editor

PLOS One

Journal Requirements:

When submitting your revision, we need you to address these additional requirements.

1. Please ensure that your manuscript meets PLOS ONE's style requirements, including those for file naming. The PLOS ONE style templates can be found at

https://journals.plos.org/plosone/s/file?id=wjVg/PLOSOne_formatting_sample_main_body.pdf and

https://journals.plos.org/plosone/s/file?id=ba62/PLOSOne_formatting_sample_title_authors_affiliations.pdf

2. Please note that PLOS One has specific guidelines on code sharing for submissions in which author-generated code underpins the findings in the manuscript. In these cases, we expect all author-generated code to be made available without restrictions upon publication of the work. Please review our guidelines at https://journals.plos.org/plosone/s/materials-and-software-sharing#loc-sharing-code and ensure that your code is shared in a way that follows best practice and facilitates reproducibility and reuse.

3. PLOS requires an ORCID iD for the corresponding author in Editorial Manager on papers submitted after December 6th, 2016. Please ensure that you have an ORCID iD and that it is validated in Editorial Manager. To do this, go to ‘Update my Information’ (in the upper left-hand corner of the main menu), and click on the Fetch/Validate link next to the ORCID field. This will take you to the ORCID site and allow you to create a new iD or authenticate a pre-existing iD in Editorial Manager.

4. If the reviewer comments include a recommendation to cite specific previously published works, please review and evaluate these publications to determine whether they are relevant and should be cited. There is no requirement to cite these works unless the editor has indicated otherwise.

[Note: HTML markup is below. Please do not edit.]

Reviewers' comments:

Reviewer's Responses to Questions

**Comments to the Author**

1. Is the manuscript technically sound, and do the data support the conclusions?

Reviewer #1: Yes

Reviewer #2: Yes

**********

2. Has the statistical analysis been performed appropriately and rigorously?

Reviewer #1: Yes

Reviewer #2: Yes

**********

3. Have the authors made all data underlying the findings in their manuscript fully available?

The PLOS Data policy

Reviewer #1: Yes

Reviewer #2: Yes

**********

4. Is the manuscript presented in an intelligible fashion and written in standard English?

Reviewer #1: Yes

Reviewer #2: Yes

**********

Reviewer #1: This work proposes AEMCF, a two‑layer adaptive framework for fuel‑cell‑battery hybrid long‑endurance UAVs. It decouples nonlinear wind‑compensating guidance from a receding‑horizon convex quadratic‑program power allocator based on equivalent‑consumption rules, avoiding solver‑model mismatch. Simulated Monte‑Carlo tests show it fully utilises onboard energy instead of stranding one power source, delivering better endurance than fixed‑ratio and offline static baselines. In all, this work seems well prepared. Below are some suggestions for further improvements:

1) Suggest integrate more advanced wind‑driven predictive forecasting techniques.

2) Suggest consider sensor imperfections. Introduce sensor bias, wind estimation drift and SOC measurement error to test real‑world robustness.

3) Guidance and power allocation are solved separately without unified optimality conditions. Suggest develop a joint optimisation formulation to capture full coupling between flight trajectory and power split.

Reviewer #2: This paper proposes AEMCF, a two-layer adaptive energy management framework for long-endurance UAVs with hybrid battery/fuel-cell power systems. The upper layer is a nonlinear, wind-compensating guidance law setting heading and airspeed, while the lower layer solves a receding-horizon quadratic program at each control step to allocate power via an equivalent-consumption principle that prevents stranding either energy source. The following issues need to be further addressed.

1. The authors should explicitly distinguish AEMCF from standard ECMS and its adaptive variants (A-ECMS). What does "wind-compensating guidance" add that a standard ECMS coupled with any flight controller cannot achieve? Without this contrast, the novelty appears incremental rather than substantive.

2. The authors should either strengthen the wind-guidance contribution or justify its inclusion with a clearer mechanism.

3. The authors should discuss the validity gap between this simulation and actual flight conditions, or provide validation against higher-fidelity models.

4. More related references should be added in this paper, for example, Joint optimisation of quality-dependent production and economic-dependent maintenance for a degradation system under online and offline inspections. International Journal of Production Research, 2026. Integrated optimization of uncertain production and scheduling-based opportunistic maintenance strategy for a multi-machine flow shop. Reliability Engineering & System Safety, 2027, 277: 113276. Joint optimization of quality control and maintenance policy for a production system with quality-dependent failures, Expert Systems With Applications, 2025, 272: 126800. Multi-level adaptive risk control: synergistic maintenance-termination optimization with online parameter learning. Reliability Engineering & System Safety, 2025: 112138. Condition-based maintenance strategy for redundant systems with arbitrary structures using improved reinforcement learning. Reliability Engineering & System Safety, 2022, 225: 108643.

**********

what does this mean?). If published, this will include your full peer review and any attached files.

If you choose “no”, your identity will remain anonymous but your review may still be made public.

**Do you want your identity to be public for this peer review?**  For information about this choice, including consent withdrawal, please see our Privacy Policy

Reviewer #1: No

Reviewer #2: No

**********

[NOTE: If reviewer comments were submitted as an attachment file, they will be attached to this email and accessible via the submission site. Please log into your account, locate the manuscript record, and check for the action link "View Attachments". If this link does not appear, there are no attachment files.]

To ensure your figures meet our technical requirements, please review our figure guidelines: https://journals.plos.org/plosone/s/figures

You may also use PLOS’s free figure tool, NAAS, to help you prepare publication quality figures: https://journals.plos.org/plosone/s/figures#loc-tools-for-figure-preparation.

NAAS will assess whether your figures meet our technical requirements by comparing each figure against our figure specifications.

# Author comment: Author response to Decision Letter 1

**DOI:** 10.1371/journal.pone.0361428.r003

3 Oct 2026

We thank both reviewers for their constructive comments. In response, we ran a new sensor-imperfection robustness experiment and used its results to substantively reframe the paper's claimed contribution: the equivalent-consumption balancing objective is robust and is now presented as the primary contribution, while the wind-compensating guidance layer is now presented as a tested, secondary feature with a small, conditionally fragile effect, rather than as an equally-weighted second pillar. We also clarified how this paper's balancing objective differs from ECMS/A-ECMS, added four of the five suggested additional references and expanded Limitations and Future Work accordingly. All simulation code and data are openly available at github.com/aeparvathy/aemcf-uav-energy. Full details are in the attached Response to Reviewers.

1. New sensor-imperfection experiment (20 seeds): new Materials and methods subsection, new Results subsection, new Table 4, new Fig 7, directly responding to Reviewer #1 Point 1.2 and contributing to Reviewer #2 Point 2.3.

2. New Related Work paragraph explicitly distinguishing AEMCF from ECMS/A-ECMS with two specific technical distinctions, responding to Reviewer #2 Point 2.1.

3. Abstract, Discussion, and Conclusion revised throughout to honestly reframe the paper's principal contribution as the resource-balancing objective, with wind-compensating guidance described as a secondary, tested, conditionally fragile feature, responding to Reviewer #2 Point 2.2.

4. Future Work expanded from four to five items, adding a concrete joint-optimisation formulation sketch (Reviewer #1 Point 1.3), advanced wind forecasting (Reviewer #1 Point 1.1), a state-estimation/sensor-fusion proposal motivated directly by the new sensor experiment, and higher-fidelity turbulence modelling and flight validation (Reviewer #2 Point 2.3).

5. 4 new references added.

6. All code and data, including both new experiments, are available at https://github.com/aeparvathy/aemcf-uav-energy.

Thank you for your valuable suggestions and efforts invested in this paper to improve the quality of the paper.

Attachment Submitted filename: Response_to_Reviewers_Round2.pdf

# Peer review: Decision Letter 1

**DOI:** 10.1371/journal.pone.0361428.r004

**Authors:** Qingan Qiu

12 Oct 2026

An adaptive energy management and control framework for long-endurance UAV missions in dynamic environments: a reproducible simulation study

PONE-D-26-43977R1

Dear Dr. Rajendran,

We’re pleased to inform you that your manuscript has been judged scientifically suitable for publication and will be formally accepted for publication once it meets all outstanding technical requirements.

Within one week, you’ll receive an e-mail detailing the required amendments. When these have been addressed, you’ll receive a formal acceptance letter and your manuscript will be scheduled for publication.

An invoice will be generated when your article is formally accepted. Please note, if your institution has a publishing partnership with PLOS and your article meets the relevant criteria, all or part of your publication costs will be covered. Please make sure your user information is up-to-date by logging into Editorial Manager at Editorial Manager® and clicking the ‘Update My Information' link at the top of the page. For questions related to billing, please contact billing support.

If your institution or institutions have a press office, please notify them about your upcoming paper to help maximize its impact. If they’ll be preparing press materials, please inform our press team as soon as possible -- no later than 48 hours after receiving the formal acceptance. Your manuscript will remain under strict press embargo until 2 pm Eastern Time on the date of publication. For more information, please contact onepress@plos.org.

Kind regards,

Qingan Qiu

Academic Editor

PLOS One

Additional Editor Comments (optional):

Reviewers' comments:

Reviewer's Responses to Questions

**Comments to the Author**

Reviewer #1: All comments have been addressed

Reviewer #2: All comments have been addressed

**********

2. Is the manuscript technically sound, and do the data support the conclusions?

Reviewer #1: Yes

Reviewer #2: Yes

**********

3. Has the statistical analysis been performed appropriately and rigorously?

Reviewer #1: Yes

Reviewer #2: Yes

**********

4. Have the authors made all data underlying the findings in their manuscript fully available?

The PLOS Data policy

Reviewer #1: Yes

Reviewer #2: Yes

**********

5. Is the manuscript presented in an intelligible fashion and written in standard English?

Reviewer #1: Yes

Reviewer #2: Yes

**********

Reviewer #1: this version is satisfactory. The comments of the reviewers are adequately addressed. The revisions are clearly highlighted.

Reviewer #2: (No Response)

**********

what does this mean?). If published, this will include your full peer review and any attached files.

If you choose “no”, your identity will remain anonymous but your review may still be made public.

**Do you want your identity to be public for this peer review?**  For information about this choice, including consent withdrawal, please see our Privacy Policy

Reviewer #1: No

Reviewer #2: No

**********

# Editor report: Acceptance letter

**DOI:** 10.1371/journal.pone.0361428.r005

**Authors:** Qingan Qiu

PONE-D-26-43977R1

PLOS One

Dear Dr. Rajendran,

I'm pleased to inform you that your manuscript has been deemed suitable for publication in PLOS One. Congratulations! Your manuscript is now being handed over to our production team.

At this stage, our production department will prepare your paper for publication. This includes ensuring the following:

* All references, tables, and figures are properly cited

* All relevant supporting information is included in the manuscript submission,

* There are no issues that prevent the paper from being properly typeset

You will receive further instructions from the production team, including instructions on how to review your proof when it is ready. Please keep in mind that we are working through a large volume of accepted articles, so please give us a few days to review your paper and let you know the next and final steps.

Lastly, if your institution or institutions have a press office, please let them know about your upcoming paper now to help maximize its impact. If they'll be preparing press materials, please inform our press team within the next 48 hours. Your manuscript will remain under strict press embargo until 2 pm Eastern Time on the date of publication. For more information, please contact onepress@plos.org.

You will receive an invoice from PLOS for your publication fee after your manuscript has reached the completed accept phase. If you receive an email requesting payment before acceptance or for any other service, this may be a phishing scheme. Learn how to identify phishing emails and protect your accounts at https://explore.plos.org/phishing.

If we can help with anything else, please email us at customercare@plos.org.

Thank you for submitting your work to PLOS One and supporting open access.

Kind regards,

PLOS One Editorial Office Staff

on behalf of

Dr. Qingan Qiu

Academic Editor

PLOS One
